# Computational and Experimental Comparison of Molecularly Imprinted Polymers Prepared by Different Functional Monomers—Quantitative Parameters Defined Based on Molecular Dynamics Simulation

**DOI:** 10.3390/molecules29174236

**Published:** 2024-09-06

**Authors:** Jing Yuan, Ying Gao, Xinzhuo Tian, Wenhao Su, Yuxin Su, Shengli Niu, Xiangying Meng, Tong Jia, Ronghuan Yin, Jianmin Hu

**Affiliations:** 1 Key Laboratory of Livestock Infectious Diseases, Ministry of Education, and Key Laboratory of Ruminant Infectious Disease Prevention and Control (East), Ministry of Agriculture and Rural Affairs, College of Animal Science and Veterinary Medicine, Shenyang Agricultural University, 120 Dongling Road, Shenyang 110866, China; jingyuan@syau.edu.cn (J.Y.);; 2 College of Sciences, Northeastern University, Shenyang 110819, China; 3 College of Information Science and Engineering, Northeastern University, Shenyang 110819, China

**Keywords:** molecularly imprinted polymers, functional monomers screening, molecular dynamics simulation, effective binding number, maximum hydrogen bond number

## Abstract

Background: In recent years, the advancement of computational chemistry has offered new insights into the rational design of molecularly imprinted polymers (MIPs). From this aspect, our study tried to give quantitative parameters for evaluating imprinting efficiency and exploring the formation mechanism of MIPs by combining simulation and experiments. Methods: The pre-polymerization system of sulfadimethoxine (SDM) was investigated using a combination of quantum chemical (QC) calculations and molecular dynamics (MD) simulations. MIPs were prepared on the surface of silica gel by a surface-initiated supplemental activator and reducing agent atom transfer radical polymerization (SI-SARA ATRP). Results: The results of the QC calculations showed that carboxylic monomers exhibited higher bonding energies with template molecules than carboxylic ester monomers. MD simulations confirmed the hydrogen bonding sites predicted by QC calculations. Furthermore, it was observed that only two molecules of monomers could bind up to one molecule of SDM, even when the functional monomer ratio was up to 10. Two quantitative parameters, namely, the effective binding number (EBN) and the maximum hydrogen bond number (HBN_Max_), were defined. Higher values of EBN and HBN_Max_ indicated a higher effective binding efficiency. Hydrogen bond occupancies and RDF analysis were performed to analyze the hydrogen bond formation between the template and the monomer from different perspectives. Furthermore, under the influence of the EBN and collision probability of the template and the monomers, the experimental results show that the optimal molar ratio of template to monomer is 1:3. Conclusions: The method of monomer screening presented in this study can be extended to future investigations of pre-polymerization systems involving different templates and monomers.

## 1. Introduction

The molecular imprinting technique (MIT) is an analytical and separation technology that artificially simulates the specific interaction between antigens and antibodies. It has enabled the development of numerous new materials for highly efficient enrichment, exhibiting exceptional performance [1,2,3,4,5,6,7,8,9,10,11,12,13]. Consequently, they are particularly well-suited for analyzing food and environmental samples that possess complex substrates.

Despite the notable advancements in research and applications of MIPs, several persistent challenges continue to hinder the rapid progress in this field. Numerous components, including the template, functional monomer, crosslinking agent, pore-causing agent, initiator, catalyst, and various parameters, such as temperature, volume, and time, are involved in the imprinting process. Any changes in the type or quantity of these raw materials may necessitate the re-optimization of the polymerization method to ensure smooth material preparation and excellent performance. Currently, polymer design largely relies on qualitative descriptions, drawing from previously accumulated experience and trial-and-error experiments. This approach demands substantial materials, energy, and particularly expensive time costs. Ultimately, the molecular mechanisms underlying the binding and recognition of MIPs with templates during the imprinting process remain unclear.

The rapid advancements in computing power and software updates have enabled the simulation of molecular imprinting systems using mathematical, chemical, and physical descriptions. Computer simulation has proven to be highly efficient in MIP design, providing accurate predictive results without relying on expensive empirical methods, such as combinatorial screening. Although the current focus on the computer-aided design of MIPs is relatively limited, QC calculations for MIP design have made significant progress [14,15,16,17,18,19,20,21]. In recent years, MD simulations have been introduced in the MIT.

Golker et al. successfully prepared MIPs predicted by MD methods, demonstrating the strong potential of MD as a tool for predicting the binding ability and affinity of templates [22]. He et al. [23] performed MD simulations and screening of an imprinting system comprising the target pollutant carbamazepine and 10 different functional monomers. Simulations and experimental data indicated the formation of a new interaction system between the template and the monomer. Energy calculations and hydrogen bond analyses suggested that the hydrogen bond between the carboxyl and amide groups, as well as the p–p conjugation of the benzene ring, significantly contributed to specific recognition. Daniels et al. reported the synthesis and evaluation of MIPs that selectively detected cortisol and studied the interaction of components in the pre-polymerization system by MD simulations. The results demonstrated that the optimal theoretical coordination of cortisol in the system occurred at a 1:6:30 component ratio of cortisol, methacrylic acid (MAA), and ethylene dimethacrylate (EGDMA), respectively. Experimental confirmation of the simulation results showcased the predictive power of these simulations [24]. 

By combining precise QC calculations with MD simulations, which are applicable to the dynamic behavior of large systems, we can gain insights into the supramolecular reactions between templates and functional monomers. Additionally, we can explore the influence of polymerization reactions on the structure of binding sites and evaluate the recognition of templates by MIPs. This approach provides a more effective means for the rational design, efficient preparation, and mechanism discovery of such materials [25,26,27,28,29,30,31,32].

This work utilized QC calculations and MD simulations to investigate the imprinting and recognition processes. The template molecule chosen was the commonly used veterinary drug SDM, while carboxylic acids and carboxylic ester functional monomers were selected. EGDMA was used as the crosslinking agent, and explicit acetonitrile served as the pore-inducing solvent. Sulfonamides were selected as template molecules because these substances represented a large number of drugs with a stable nuclear parent and regular side-chain changes. That was, the substituents on the sulfonamide group were mostly six-membered or five-membered heterocyclic rings. The substituent of SDM was 2, 6-dimethoxy-4-pyrimidine, which was large in size and had many hydrogen bonding sites. Drugs are well known to have a definite structure–activity relationship. Thus, MIPs should also have a similar structure–selectivity relationship. Exploring the molecular mechanisms of imprinting and recognition from the perspective of structural differences, as well as discovering the rules affecting selective recognition, is a worthwhile undertaking. 

From the perspective of the molecular level, our work aims to clarify the interaction mode among major components, such as functional monomers, template molecules, and crosslinkers, by transforming the types of functional monomers and the number of monomers interacting with the template, identifying a variety of sulfonamides, etc., so as to consider the binding efficiency of different functional monomers and template molecules. The calculation data were validated through preparation and adsorption tests. To our knowledge, few reports have attempted to use MD simulation to propose quantitative evaluation criteria for the imprinting effect between templates and functional monomers. The results and methods presented in this paper are expected to contribute to the rational design of MIPs for sulfonamide identification in the future and can be extended to the investigation of pre-polymerization systems involving diverse templates and monomers.

## 2. Results and Discussion

### 2.1. QC Calculations

The binding sites within the matrix significantly influence the recognition properties of MIPs. Computational techniques are crucial in MIP design and have been extensively utilized to investigate the molecular-level intermolecular interactions between templates and functional monomers. In this study, the template and monomer conformations were optimized using the B3LYP level with the 6-31G(d) basis set. Following optimization, a natural bond orbital (NBO) analysis was performed to examine the charge characteristics of atoms functioning as hydrogen bond donors and acceptors [33].

The structural parent core of sulfonamides is p-aminobenzene sulfonamide. The imprinted group present in these veterinary drugs consists of a primary amino group and an imide group, which provide three hydrogen bond donors, as well as a sulfonyl group that offers two hydrogen bond acceptors (See Table S1). Furthermore, different drugs feature varying side chains attached to the parent nucleus, which may include additional groups that can form hydrogen bonds, such as heterocyclic nitrogen or a methoxy group.

Functional monomers can generally be categorized into two types. One type includes monomers that possess a hydrogen bond acceptor and a hydrogen bond donor. Examples of such monomers are carboxylic acid and acrylamide monomers. Carboxylic acid functional monomers, for instance, contain an acceptor group, oxygen (=O), and a donor group, hydroxyl (-OH), allowing the monomers to engage in diverse interactions with other molecules (Table S1). However, self-aggregation between monomers can occur. Figure S1 illustrates the structures of the 1:1 complexes between SDM and acrylic acid (AA), naming them SDM-AA① to SDM-AA⑧. These were the eight possible scenarios for the interaction between SDM and AA found by QC calculations. The binding energy in vacuum ∆*E*_bind_, the hydrogen bond length, and the angle of different 1:1 template–monomer complexes (Table 1) were calculated to analyze the interactions between the monomer and the template in detail. The hydrogen bond donor and acceptor of AA are adjacent in space. Hence, double hydrogen bonds can be formed to make the binding between the template and the functional monomer more stable. For instance, SDM-AA③ formed double hydrogen bonds of N-H⋯O=C and S=O⋯H-O, and ∆*E*_bind_ is −68.12 kJ/mol. Compared with the N-H⋯O=C of SDM-AA①, ∆*E*_bind_ was −30.17 kJ/mol. SDM-AA⑤ generated N-H⋯O=C and pyrimidine para-N⋯H-O double hydrogen bonds with a ∆*E*_bind_ of −82.30 kJ/mol, thereby enabling higher energy than SDM-AA③ due to the presence of p–π conjugate. The combination of MAA, 4-vinylbenzoic acid (4-VBA), and trifluoromethylacrylic acid (TFMAA) with SDM was similar to AA (the binding sites and naming principle were the same as SDM-AA complexes). The ∆*E*_bind_ of SDM-TFMAA⑤, SDM-4-VBA⑤, and SDM-MAA⑤ were −91.63 kJ/mol, −83.30 kJ/mol, and −84.50 kJ/mol, respectively. The side chain of AA does not contain any power supply or absorbent groups. Meanwhile, the side chains of MAA and 4-VBA serve as power-supply groups, whereas the side chain of TFMAA acts as an absorbent group. According to the data in Table S1, the charges of the hydroxyl groups of the four functional monomers were almost the same. The presence of side chains mainly affected the charge of carbonyl oxygen. Obviously, the carbonyl oxygen of TFMAA had the weakest electronegativity, while the ∆*E*_bind_ of SDM-TFMAA⑤ was the highest. The reason is that the F atom on the side chain is close to the benzene ring of SDM in space and can form weak hydrogen bonds with C-H bonds (Figure S2). Therefore, when screening or designing functional monomers, if there are other highly electronegative atoms near the hydrogen bond donor/acceptor of the monomer, it is also possible to form strong/weak hydrogen bonds with the template molecule, which will improve the stability of the complex.

Another category of functional monomers consists of those that possess either hydrogen bond acceptors or hydrogen bond donors exclusively. Examples of such monomers include carboxylic ester monomers and vinylpyridine. These monomers form single-type interactions with other molecules and do not undergo self-polymerization. Carboxylic ester functional monomers, such as 2-ethylhexyl methacrylate (EHMA), ethyl methacrylate (EMA), and methyl methacrylate (MMA), contain two distinct types of hydrogen bond acceptors, namely, the carbonyl oxygen (=O) and the ester oxygen (-O-). These monomers have fewer binding sites (Figure S3) and exhibit lower binding energies than carboxylic acid monomers (Table 1).

Based on energy considerations and the abundance of binding sites, carboxylic acid functional monomers outperform acrylate monomers in the synthesis of MIPs.

### 2.2. MD Simulations

**Figure 1:**
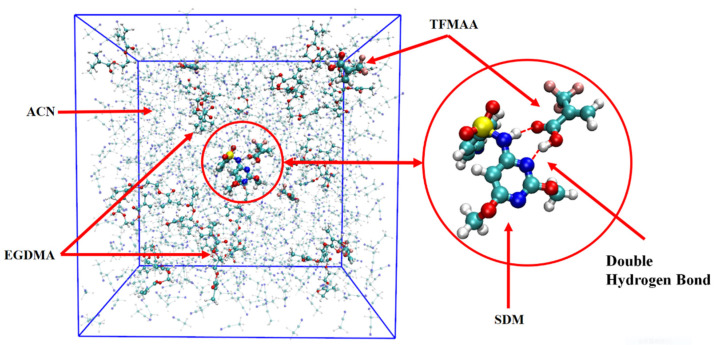
Frame simulation diagram in the TFMAA polymerization system (the left side is the complete system, and the right side is the template and monomer that form the double hydrogen bond after local amplification. The pre-polymerization system is composed of SDM:TFMAA:EGDMA = 1:8:40).

During the production-phase simulation, the trajectory was recorded every 500 frames, resulting in a total of 20,000 frames capturing the system’s structural evolution. Observing the animation of the complete trajectory, the template molecules and functional monomers continuously associate and dissociate. This behavior, as highlighted by Kouki et al. [34], indicates that these interactions are typically weak and reversible, resulting in a rapid kinetic equilibrium between the complexed and free molecules. Figure 1 displays a specific snapshot from the trajectory, showcasing the structural arrangement at that particular moment.

#### 2.2.1. The Effective Binding Number (EBN) and the Maximum Hydrogen Bond Number (HBN_Max_)

Combined with the results of the QC calculations above, we observe how many functional monomers SDM can bind to in each frame of the MD simulations and at which hydrogen bond sites are they bound. 

The results showed that the template–monomer complexes appearing in the trajectories generated by MD simulations were formed according to the hydrogen bonding binding sites predicted in the QC calculations. The complexes did not exist stably after generation but were constantly dissociated and formed. They could be formed with a molar ratio of functional monomer to template molecule of 1:1, including those bound by single or double hydrogen bonds. Of note, the N-H⋯O=C and pyrimidine para-N⋯H-O double hydrogen bonds formed by carboxylic acid monomers mentioned in the QC calculations, like that of SDM-AA⑤, occurred more frequently than the N-H⋯O=C and S=O⋯H-O double hydrogen bonds, like that of SDM-AA③. In addition, there were also complexes formed with a molar ratio of 2:1 between functional monomer and template molecule, including those with two single hydrogen bonds or three hydrogen bonds (one double hydrogen bond and one single hydrogen bond). 

In other words, when the molar ratio of functional monomer to template molecule was up to 10:1, at most two monomers could simultaneously form hydrogen bonds with SDM. This finding suggested that the sites for simultaneous hydrogen bonding with SDM were limited, even when a large number of monomers were present in the system. Engrid Juni Astuti et al. [35] also observed that as the ratio of monomer to template increased from 1 to 10, the average number of hydrogen bonds in the system remained at 3–4. This phenomenon also indirectly proved this point. T Sajini et al. [17] reported that the most appropriate L-phenylalanine benzyl ester-to-MAA ratio was determined to be 1:2 through QC calculation and experimental verification.

To evaluate the binding efficiency of the functional monomers and the template molecules, we defined two parameters, namely the effective binding number (EBN) and the maximum hydrogen bond number (HBN_Max_). The EBN represents the maximum number of functional monomer molecules that can bind to a template molecule, while HBN_Max_ represents the maximum number of hydrogen bonds that can be formed between the functional monomers and the template. Table 2 shows the EBN and HBN_Max_ values between several monomers and SDM. Since carboxylic acid monomers can form double hydrogen bonds with SDM, inconsistencies may arise between EBN and HBN_Max_ values. Higher values of EBN and HBN_Max_ indicate a higher effective binding efficiency of the functional monomers and the template. 

#### 2.2.2. Analysis of Hydrogen Bond Occupancy

**Figure 2:**
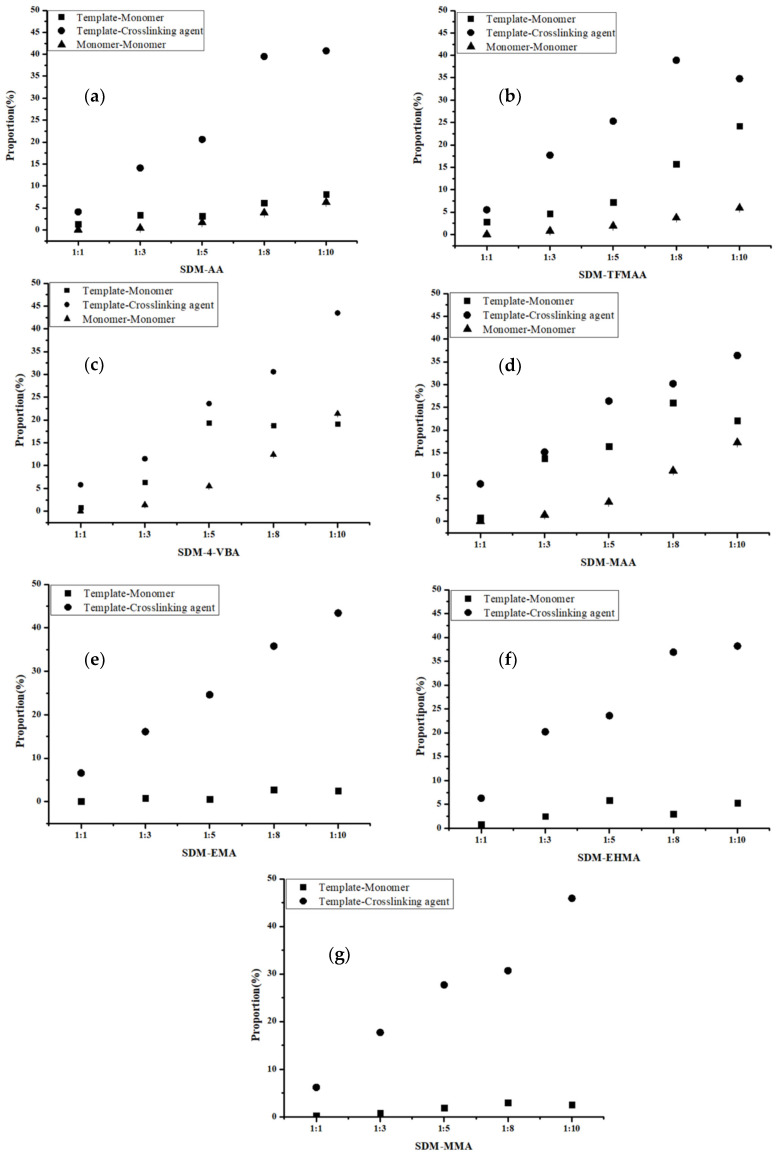
Analysis of hydrogen bond occupancy generated by different molar ratios of SDM to functional monomers ((**a**): AA, (**b**): TFMAA, (**c**): 4-VBA, (**d**): MAA, (**e**): EMA, (**f**): EHMA, (**g**): MMA). Since the carboxylic ester monomers have only hydrogen bond acceptors and no hydrogen bond donors, self-polymerization cannot occur. So, the last three figures (**e**–**g**) do not have data for the “monomer-monomer” group.

**Figure 3:**
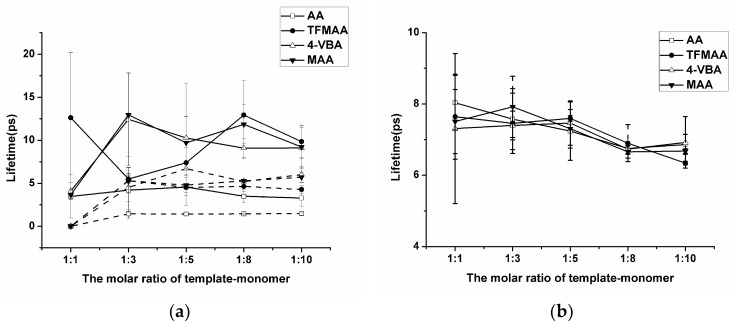
Analysis of the lifetime of the hydrogen bond generated by SDM to functional monomers or crosslinkers. (In (**a**), “solid line” represents template–monomer and “dash line” represents the monomer–monomer; In (**b**), “solid line” represents template–crosslinker).

The “Hydrogen Bonds” plug-in of VMD is used to calculate hydrogen bond occupancy, which represents the average number of hydrogen bonds occurring per frame between the hydrogen bond donor and acceptor in the selected trajectories. The higher the hydrogen bond occupancy, the more stable the hydrogen bond is. Figure 2 demonstrates that, as the amounts of functional monomers increase, the likelihood of generating hydrogen bonds through collisions with template molecules also increases, leading to higher hydrogen bond occupancy in a complex formation. When carboxylic acid monomers were used, the increase in hydrogen bond occupancy was great, while the increase was relatively small when ester monomers were used. Ester monomers generally exhibited weaker hydrogen bonding abilities. Notably, carboxylic acid monomers can self-aggregate due to the presence of hydrogen bond donors and acceptors, which has raised concerns about their imprinting ability among researchers [36]. Regarding hydrogen bond occupancy, the incidence of monomer dimers is indeed relatively high, but it generally remains lower than the hydrogen bond occupancy between template molecules and functional monomers. We also calculated the lifetime of hydrogen bonds in the system (Figure 3a) and found that the lifetime of hydrogen bonds generated during self-polymerization is shorter than that between template molecules and functional monomers. 

In addition, the number of hydrogen bonds between the crosslinker and the template molecule is greater compared to those between the template molecule and the functional monomer. The main reason is that the amount of crosslinker is large, and it is a component that cannot be ignored in the molecular imprinting system. However, in pre-polymerization systems with different functional monomers, the degree of complexation between the crosslinking agent and the template molecule remains relatively similar. Golker et al. [22] also observed that EGDMA interacted with the template through hydrogen bonds for more than 45% of the total simulation time. However, these hydrogen bond contacts were notably unstable, and the average lifetime of these interactions was short. As shown in Figure 3b, the lifetime of the hydrogen bond between the template and the crosslinker does not vary much when different functional monomers are replaced, but it may be longer or shorter than the lifetime of the hydrogen bond between the template and the functional monomer.

#### 2.2.3. Radial Distribution Function (RDF) Analysis

**Figure 4:**
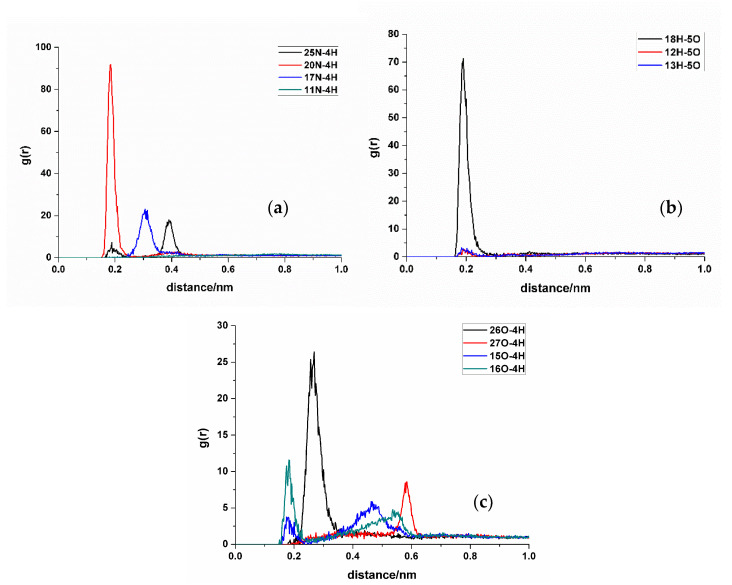
RDF analysis of TFMAA and SDM binding sites. (**a**,**b**): hydrogen bond acceptor of SDM to hydrogen bond donor of TFMAA; (**c**): hydrogen bond donor of SDM to hydrogen bond acceptor of TFMAA.

**Figure 5:**
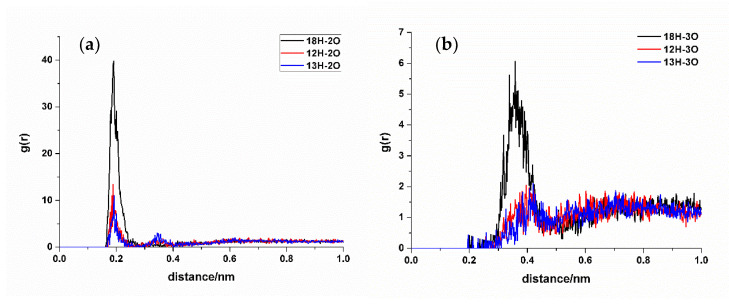
RDF analysis of EHMA and SDM binding sites. (Hydrogen bond donor of SDM to hydrogen bond acceptor ((**a**): carbonyl oxygen, (**b**): ester oxygen) of EHMA).

In RDF analysis, the reference atom Ai is set as the hydrogen bond atoms of SDM, while the statistical atom Bj represents the hydrogen bond atoms of the functional monomer. The calculated results show that the density of Bj in the spherical shell with a radius of r from Ai is relative to the average density of Bj in the entire system. RDF analysis can provide insights into the frequency of hydrogen bond formation sites between the two molecules and the distances between the atoms involved in the hydrogen bonds in the condensed phase. Different hydrogen bond formations correspond to the varying atomic distances between different atoms. A shorter distance corresponds to a smaller hydrogen bond length, whereas a higher peak height means higher hydrogen bond formation frequency. Taking TFMAA, an acrylic acid monomer, as an example (Figure 4), the most frequently generated hydrogen bonds are 18H-5O, 20N-4H, and 26O-4H. Notably, 18H-5O and 20N-4H correspond to the double-bond binding sites described in Figure 1. Examining EHMA, an ester monomer, as an example (Figure 5), the image indicates that the binding frequency between 18H and 2O, as well as 3O oxygen, is higher than that of the primary amino group. In different ratios, the binding frequencies of one or two primary amine hydrogens and imide hydrogens are similar. Additionally, the binding frequency with oxygen number 2 (carbonyl oxygen) is higher than that with the hybrid ester oxygen of number 3 (sp3 oxygen). All the 1:1 complexes of SDM and EHMA found during QC calculations are bound to SDM by oxygen number 2, which is consistent with the results of RDF analysis. By looking at the structure of the 1:1 complexes, it can be found that there are no other atoms around the oxygen number 2, and the position is prominent. Meanwhile, the oxygen number 3 is blocked by other atoms around and has steric hindrance, so it is not easy to interact with the hydrogen atom of SDM. Similar conclusions can be drawn for other ester monomers. Comparing the data of carboxylic acid monomers and ester monomers, the formation frequency of hydrogen bonds in carboxylic acid monomers is generally higher than that in ester monomers. The EBN of both TFMAA and EHMA is two, that is, two monomers can interact with the template at the same time. However, the HBN_Max_ of TFMAA is three, which is larger than that of EHMA. The complex of TFMAA and SDM can produce three hydrogen bonds with lower energy, and it can be clearly found that this complex occurs more frequently through RDF analysis. Therefore, it is more reliable to evaluate the interaction ability of the template and the monomer by both EBN and HBN_Max_ parameters.

### 2.3. Synthesis and Characterization of Surface MIPs (SMIPs)

In this study, SI-SARA ATRP technology was used to prepare SDM-SMIPs, which was the same as the previous study [21]. 

**Figure 6:**
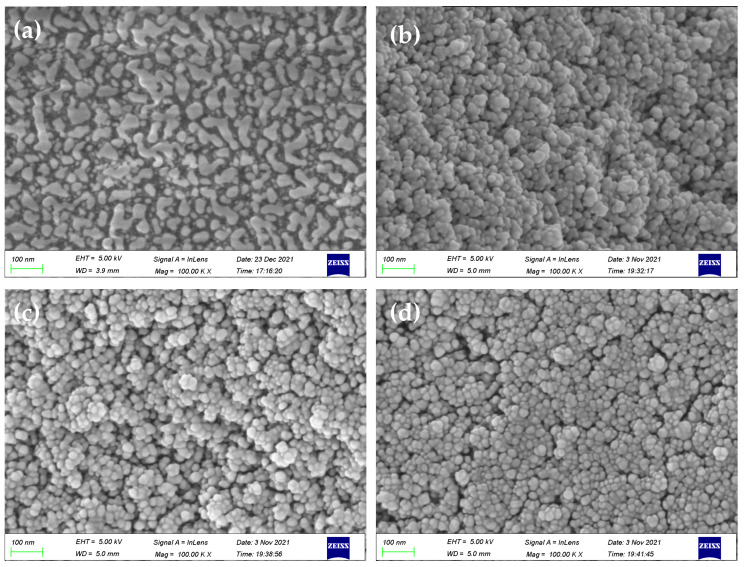
SEM images of SiO_2_@Br (**a**) and SiO_2_@MIP prepared by AA, TFMAA, and 4-VBA (**b**–**d**).

SEM images (Figure 6) exhibited the presence of delicate protrusions on the surface of silica gel. In comparison with initiator-functionalized silica gel (SiO_2_@Br), the SMIPs synthesized using AA, TFMAA, and 4-VBA displayed numerous densely packed spherical particles on the microsphere’s surface. The surface structure appeared thicker, scattered, and undulating, thereby enhancing the three-dimensional appearance. These images indicate the successful formation of a uniform molecularly imprinted layer on the surface of SiO_2_ microspheres, confirming the successful polymerization process.

Figure S4 displays the thermogravimetric curves of the products at various stages. Following elution, the mass losses observed for AA, TFMAA, and 4-VBA were 37.5%, 14%, and 24.1%, respectively. The thickness of the imprinted shell formed on the silicon surface could be ordered as follows: AA > 4-VBA > TFMAA. These results demonstrated that all of the reactions had successfully occurred under the same experimental conditions. However, the difficulty of reaction of each monomer differed.

### 2.4. Evaluation of the SMIPs

#### 2.4.1. Effect of the Functional Monomer Species on the Adsorption Effect

**Figure 7:**
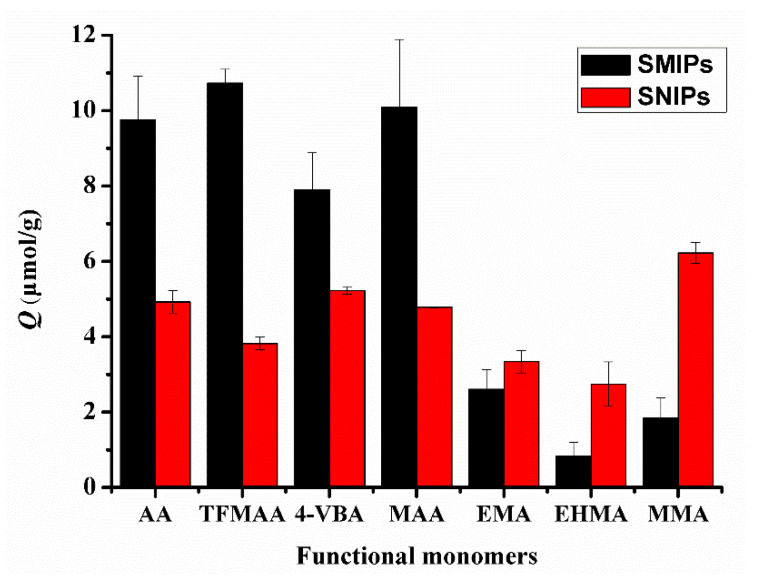
Comparison of the adsorption capacities of SMIPs and SNIPs synthesized by carboxylic acid and ester functional monomers. (SDM:monomers = 1:3).

In Figure 7, the adsorption capacity of SMIPs synthesized using carboxylic acid monomers, such as AA, TFMAA, 4-VBA, and MAA, was higher than that of SMIPs synthesized using ester monomers that only possess hydrogen bond receptors, including EMA, EHMA, and MMA. This observation aligns with the conclusions obtained from the previous simulation calculations. Additionally, a similar procedure without the addition of a template was used to prepare surface non-imprinted polymers (SNIPs). The adsorption capacity of SMIPs synthesized with carboxylic acid monomers exceeded that of SNIPs, indicating their selectivity for the template molecules. Meanwhile, the adsorption capacity of SMIPs synthesized with ester monomers was even lower than that of SNIPs, indicating poor selectivity. Among the functional monomers, TFMAA exhibited the highest energy and superior imprinting effect compared with others. Conversely, 4-VBA demonstrated the lowest adsorption capacity, possibly due to the presence of a benzene ring in its molecular structure, which may contribute to steric hindrance. 

#### 2.4.2. Effect of the Amount of Functional Monomers on the Adsorption Effect

**Figure 8:**
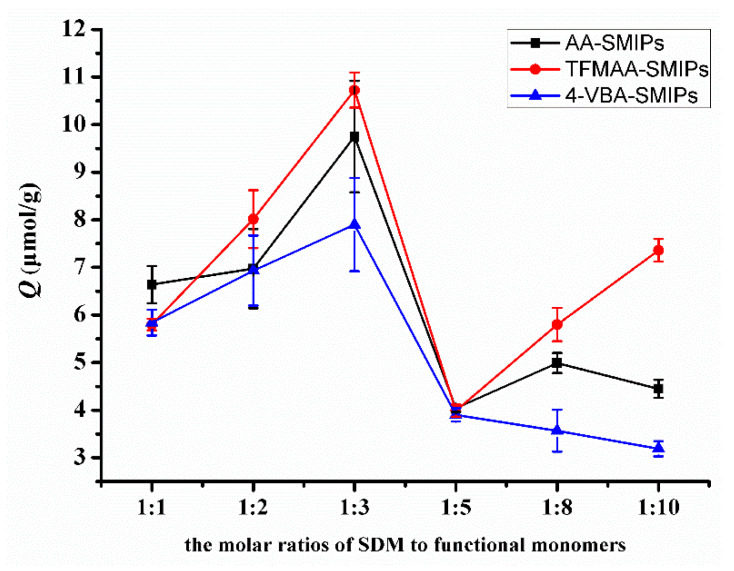
Comparison of the adsorption capacities of SMIPs synthesized by AA, TFMAA, and 4-VBA in different molar proportions.

In Figure 8, the adsorption amount exhibits a peak when the molar ratio of template molecule to functional monomer is 1:3. Theoretically, the imprinting sites reach a saturation state when the molar ratio is 1:2. However, with fewer monomers, the likelihood of collisions with the template molecule to form hydrogen bonds is lower. Increasing the number of monomers enhances the probability of hydrogen bond formation but also leads to an increase in non-specific binding. Previous research [21,35] has indicated that an excessive amount of functional monomers tightly wraps around the template molecule, creating greater steric resistance when the template molecule enters or exits the pores. This situation hinders the elution and reabsorption of the template molecule. Therefore, the molar ratio of 1:3 becomes the most suitable ratio.

#### 2.4.3. Binding Specificity

**Figure 9:**
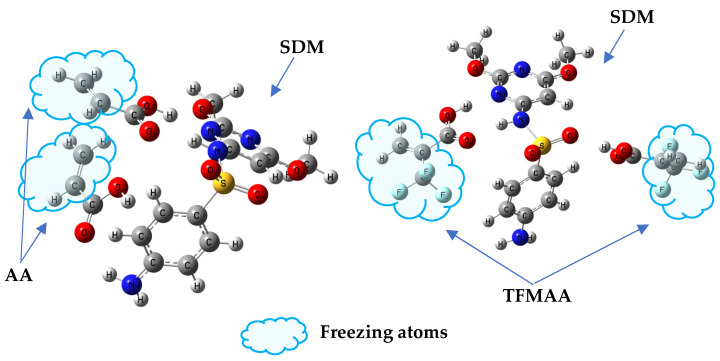
“Freeze” the cross-linked structure of the monomer to simulate “holes”. Replacement of SDM with other drug molecules could be used to model selective adsorption. (**left**: SDM-AA 1:2 complex; **right**: SDM-TFMAA 1:2 complex).

**Figure 10:**
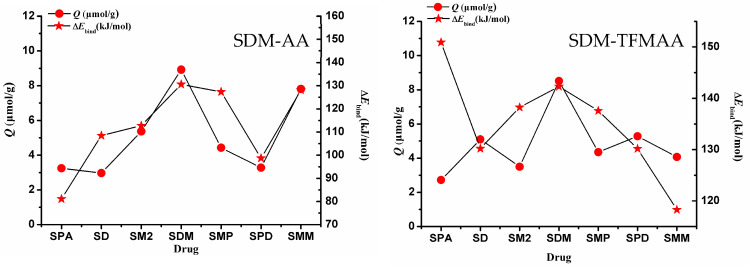
Adsorption amounts and weak interaction energies of the two SMIPs on seven sulfonamides. (**left**: SDM-AA; **right**: SDM-TFMAA).

To investigate the impact of weak interaction energy on the selective adsorption of MIP, a restricted optimization QC method was employed to simulate the interaction between “pores” and various sulfonamides. The process involved optimizing the structure of the SDM-functional monomer complex with a 1:2 ratio, based on the results of MD simulation (Figure S5). Following optimization, the SDM molecules were removed, and the cross-linked atoms in the functional monomer molecules were frozen to simulate the “holes” after elution (Figure 9). These holes were then combined with other sulfonamides to simulate re-adsorption. Figure 10 illustrates the relationship between the intermolecular interaction energy of sulfonamides and the two SMIP-imprinted holes obtained through computer simulation and the Q (adsorption capacity) obtained in the experiment. Table S2 presents the chemical structures of the drugs (sulfapyridine (SPD), sulfadiazine (SD), sulfamethazine (SM2), sulfamonomethoxine (SMM), sulfaphenazole (SPA), and sulfamethoxypyridazine (SMP)). 

When considering only the calculated binding energy, it is evident that the binding energy varies for the same drug with different holes. For instance, the energy of SPA combined with AA-SMIPs is minimal, whereas the energy of SPA combined with TFMAA-SMIPs is maximal, exceeding even the energy of SDM combined with the holes. The reason behind this discrepancy lies in the fact that SPA can only form two individual hydrogen bonds when interacting with AA-SMIPs (see Figure S6). In other words, the HBN_Max_ is two, not three. However, when combined with TFMAA-SMIPs, an additional weak hydrogen bond is formed (see Figure S6). This information is not readily apparent from the two-dimensional or even three-dimensional structure of the drug alone, necessitating the use of calculations to discern these details.

The experimental results of adsorption capacity for AA-SMIPs align well with the calculated weak interaction-energy data. However, the degree of agreement between the two sets of data for TFMAA-SMIPs is not as strong, particularly in the case of SPA mentioned earlier. Although SPA exhibits a high weak interaction energy when combined with the holes, the measured adsorption capacity in the experiment is very low. This discrepancy can be attributed to steric hindrance. While our simulation involves directly placing drug molecules into the holes for structure optimization, in reality, the drugs must find a way to enter the holes and bind to the binding sites. Steric hindrance can arise from two factors. First, the three-dimensional structure of the monomer plays a role. For example, TFMAA used in this experiment contains an additional trifluoromethyl group compared to AA. Second, the three-dimensional structure of the drug itself, such as the side chain of SPA’s nitrogen heterocyclic ring, which includes a benzene ring, can contribute to steric hindrance.

## 3. Materials and Methods

### 3.1. Chemicals and Materials

Silica gel (360 mesh, irregular shape) and copper(II) bromide (CuBr_2_, >99%) were purchased from Shanghai Titan Scientific Co., Ltd. (Shanghai, China); 3-Aminopropyltriethoxysilane (APTES, 99%), 4-VBA (97%), EGDMA (98%), MAA (>99%), SDM (98%), SPD (98%), SD (98%), SM2 (99%), SMM (>93%), SPA (≥98%), and TFMAA (98%) were obtained from Aladdin Industrial Corporation (Shanghai, China). Iron powder (Fe(0), 99%) was from Shanghai Macklin Biochemical Co., Ltd. (Shanghai, China). *N*,*N*,*N*′,*N*″,*N*″-Pentamethyldiethylenetriamine (PMDETA, >98%), 2-Bromoisobutyryl bromide (BIBB, 98%), and SMP (>98%) were purchased from TCI Shanghai (Shanghai, China); AA (99%), and EHMA (98%), MMA (99%), EMA (99%) were purchased from Sigma-Aldrich (Missouri, USA). All the reagents were of analytical grade. Self-prepared deionized water with a resistivity of 18.2 MΩ/cm was used to prepare all the solutions. All the experiments were performed at least three times.

### 3.2. Instruments and Software

The bromine content in initiator-functionalized silica gel was determined by oxygen bomb combustion (PARR-6400, Parr Instrument Company, Moline, IL, USA) and the ion chromatography (Dionex ICS-3000, Thermo Fisher Scientific, Waltham, MA, USA) (OBC-IC) method. The polymers were coated with platinum under a vacuum by an ion sputter, which was then used for morphology observation by scanning electron microscope (SEM) (Sigma300, Zeiss, Oberkochen, Germany). The thermal degradation of the prepared MIPs was investigated using a thermogravimetric analyzer (TGA) (7200, Hitachi, Tokyo, Japan). The measurement was carried out from room temperature to 800 °C at a heating rate of 10 °C/min under a N_2_ atmosphere. Ultrapure water was prepared by a Nanopure water purifier (NANOpure DIamond, Thermo Fisher Scientific, USA). Computer simulations were carried out via Gaussian 09 [37], GROMACS 2018 [38,39] and Multiwfn 3.8(dev) [40].

### 3.3. Chromatographic Conditions

The high-performance liquid chromatography (HPLC) analysis system was a Dionex Ultimate 3000 HPLC System (Thermo Fisher Scientific, USA) equipped with a quaternary pump, on-line degasser, six-port injection valve, column thermostat, and UV–vis detector. Data acquisition and integration were performed using Chromeleon 7.0 series software from Dionex. For the chromatographic column, Venusil XBP C18 was 4.6 mm × 150 mm, 5 μm (Dikma technologies, Beijing, China). The column temperature was 30 °C. The detection wavelength was 272 nm. The mobile phase was an acetic acid solution (2%)—acetonitrile (65:35, *V*/*V*). The flow rate of the mobile phase was 1 mL min^−1^. The injection volume was 20 μL.

### 3.4. Molecular Modeling

The QC calculations were calculated using Gaussian 09 software. In detail, geometry optimization was performed by applying the DFT method at the B3LYP level with the 6–31G(d) basis set, and vibrational frequency calculations were conducted to verify the true minima. DFT at the M06-2X level with a 6-311+G(d,p) basis set was used to calculate the single-point energy and binding energies between the template and functional monomers. Basis set superposition error (BSSE) was included in the total interaction energy, and components calculations were carried out using the counterpoise (CP) method. The ∆*E*_bind_ was calculated using Equation (1) [41].

∆*E*_bind_ = *E*_complex_ − *E*_template_ − ∑*E*_monomer_ (1)

where *E*_complex_ was the potential energy of the template–monomer complex, *E*_template_ was the potential energy of the template, and ∑*E*_monomer_ was the total potential energy of the functional monomers.

The MD simulations were performed using the GROMACS 2018 software. The topologies for the molecules were generated using acpype/antechamber (v. 0 0 Rev: 0) [42,43] and were based on the general amber force field. The RESP2(0.5) charge was calculated using Gaussian combined with Multiwfn 3.8(dev). In the simulations, the SDM molecule was positioned at the center of the cubic box, whereas the functional monomers were placed in a spherical region around the SDM. The outer layer of the box was filled with EGDMA, and the remaining space in the cubic box was randomly filled with acetonitrile molecules. This was consistent with the order in which the reagents were added in the experiment. First, the previous molecules were energy-minimized, with 10,000 conjugate gradient steps to remove bad contacts. Then, the pre-polymerization system was gradually heated to 333.15 K during a 200 ps time under conditions of an NPT ensemble using a Berendsen pressure bath and a velocity-rescale hot bath with the pressure set to 1 atm. Finally, a production-phase simulation of 20 ns was carried out under conditions of an NPT ensemble using a Parrinello–Rahman pressure bath and velocity-rescale hot bath with the temperature set to 333.15 K and pressure to 1 atm. The coordinates and energy were stored every 500 steps with the time step of 2 fs. In these simulations, periodic boundary conditions were employed together with a 10 Å nonbonded interaction cutoff. The particle mesh Ewald (PME) method was used to calculate the electronic interaction among the molecules in the system, and the cut-off method was used to calculate the van der Waals effect. All hydrogen atoms in the system were constrained using the LINCS algorithm. The analysis tools for studying the energy and trajectory of the simulated system were facilitated with the GROMACS software package and visual molecular dynamics (VMD 1.9.3) [44]. The in-built GROMACS tools were used for the calculations of weak interaction energy, the assessment of radial distribution functions (RDFs), and the analysis of H-bonds. When calculating the weak interaction, the trajectory is rerun. The cut-off method was used to calculate the electronic interaction and the van der Waals effect with a cut-off of 2.0 nm. In the hydrogen bond analysis, the upper limit of the distance between the donor and acceptor atoms is 0.35 nm, and the upper limit of the angle between the hydrogen donor–acceptor is 30 degrees [45,46].

### 3.5. Synthesis of SMIPs

**Figure 11:**
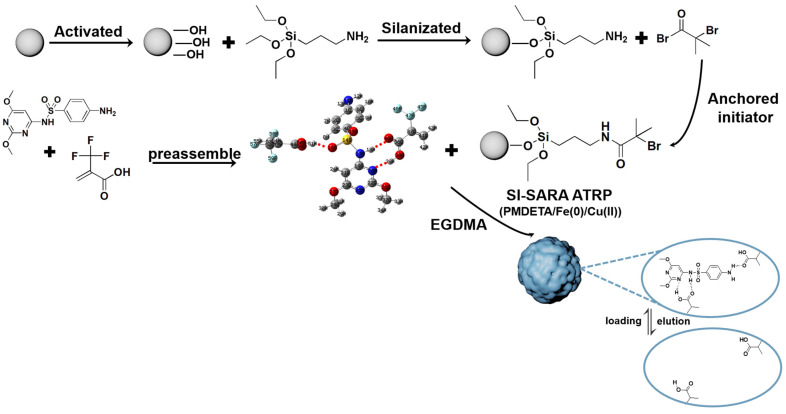
The synthetic route for SMIPs, taking TFMAA as a functional monomer for example.

A SI-SARA ATRP method, tolerant to air, was utilized for the preparation of SMIPs (Figure 11). This approach used Fe(0)/Cu(II) as the catalytic system and silica gel as the core material [21]. SARA ATRP is a highly reliable and user-friendly ATRP technique [47] that does not necessitate the use of air-sensitive materials or metal salts [48]. 

To prepare the silica gel for grafting, a pretreatment method described in the previous literature [49] was used to eliminate surface contaminants and activate the surface silanol groups. The pretreated silica gel was then subjected to silanization by adding 5% APTES (*w*/*w*) to the silica gel suspension in anhydrous ethanol. The suspension was stirred for 24 h at room temperature under a nitrogen atmosphere. The grafted silica gel was subsequently filtered, washed with methanol multiple times, and dried in a vacuum at 60 °C. The anchoring of the initiator was carried out as follows. First, 5 g of amine-functionalized silica gel (SiO_2_@NH_2_) and 0.35 mL of TEA were mixed in 45 mL of dichloromethane at 0 °C. Then, the dropwise addition of a solution containing 0.391 g of BIBB in 5 mL of dichloromethane was performed under a nitrogen atmosphere. The resulting suspension was stirred for 1 h at 0 °C, followed by another 23 h at room temperature. The SiO_2_@Br was filtered, washed thoroughly with water, and dried in a vacuum at 60 °C. For the preparation of SMIPs, a typical SI-SARA ATRP reaction procedure was followed [42]. Initially, the template (0.417 mmol) was weighed and dissolved ultrasonically in a three-necked flask with 15 mL of acetonitrile. Then, 1.0 g of initiator-functionalized silica gel was added to the flask and stirred at room temperature. The appropriate amount of monomer was dissolved separately in 5 mL of acetonitrile in a small beaker, ultrasonicated for 20 min, and transferred to the flask. After stirring at room temperature for 1 h, the EGDMA (cross-linker), in an amount five times that of the monomer, was added to the flask, and the mixture was stirred for 1 h. Subsequently, PMDETA (0.25 mmol) and CuBr_2_ (0.125 mmol) were added to the mixture and stirred for dissolution. Finally, under anaerobic conditions, iron powder (0.625 mmol), as the reducing agent, was added to the flask. The flask was sealed, and polymerization was initiated in a water bath at 80 °C with magnetic stirring for 4 h (the aim is to produce enough Cu(I)), followed by cooling to 60 °C for 20 h. The product was washed alternately with acetonitrile and methanol–acetic acid (9:1, *v*/*v*) via the soaking method at 50 °C, as well as solid-phase extraction, until the washing solution was free of SDM, as confirmed by HPLC. Finally, the SMIPs were dried in a vacuum at 60 °C. A similar procedure, without the addition of a template, was used to prepare SNIPs as the control materials.

### 3.6. Binding Experiments

Binding experiments were conducted at 25 °C with continuous shaking. A total of 30 mg of SMIPs or SNIPs were weighed and added to 3 mL of dichloromethane solutions containing SDM or other sulfonamides. The mixtures were then shaken for 2 h. The drug concentration in the filtrate was measured using an HPLC analysis, and the amount of drug bound to the polymers (Q, in μmol g^−1^) was determined as the difference between the initial and final drug concentrations in the solutions.

## 4. Conclusions

Molecular imprinting systems are characterized by their complexity and diversity, presenting intriguing concepts and properties that have captivated the interest of numerous researchers. These researchers continuously strive to determine which monomers should be selected for a specific target, what substances can be selectively adsorbed by MIPs, and how efficiently they can be adsorbed. Despite having a series of drugs with similar functional atoms or groups, such as sulfonamides, each template molecule possesses its own unique characteristics. The differences in the three-dimensional structure among these analogs give rise to distinct behaviors during the imprinting and adsorption processes, which are even more intricate than anticipated. To unravel this complexity, the combined use of QC calculations and MD simulations can provide valuable insights.

This study focuses on the MD simulation of the pre-polymerization system. The findings reveal that, within a certain range of proportions, only two carboxylic acid or carboxylic ester functional monomers can simultaneously bind to the template molecule SDM at most. The identified hydrogen bond binding sites align with the predictions obtained from the results of the QC calculations. The EBN and HBN_Max_ were introduced as measures to evaluate the binding efficiency of the functional monomers. The constant combination and dissociation of SDM with various carboxylic acids and carboxylic ester monomers indicate the challenge of forming stable associations, possibly due to the weak interaction forces between the components. An analysis of the hydrogen bond occupancy and the RDF supports the conclusion that carboxylic acid functional monomers exhibit better imprinting effects compared to ester monomers. Furthermore, under the influence of the EBN and collision probability of template molecules and functional monomers, the experimental results show that the optimal molar ratio of the template molecule to the functional monomer is found to be 1:3.

The rational selection of monomers plays a critical role in enhancing the adsorption performance of MIPs. Utilizing computer simulation to identify more suitable functional monomers is an effective approach. The integration of QC calculations and MD simulations, as described in this paper, holds great promise in this regard. It offers a range of possibilities for improving the design and performance of MIPs.

## Figures and Tables

**Table 1:** Interaction energies and hydrogen bond parameters of SDM with AA or EMA.

1:1 Template-Monomer Complexes	Hydrogen Bond Number	Δ*E*_interation_ (kJ/mol)	Hydrogen Bond * (Template⋯Monomer)	Charge (Acceptor⋯Donor)	Bond Length (nm)	Bond Angle (°)
SDM-AA①	1	−30.17	N-H⋯O=C	−0.643⋯0.422	0.2023	164.9
SDM-AA②	1	−33.19	N-H⋯O=C	−0.645⋯0.424	0.2033	156.3
SDM-AA③	2	−68.12	N-H⋯O=C	−0.665⋯0.471	0.1928	164.5
			S=O⋯H-O	−0.975⋯0.534	0.1758	172.4
SDM-AA④	1	−48.83	S=O⋯H-O	−0.978⋯0.532	0.1818	173.4
SDM-AA⑤	2	−82.30	N-H⋯O=C	−0.670⋯0.469	0.1843	169.6
			Pyrimidine para-N⋯H-O	−0.639⋯0.531	0.1786	176.4
SDM-AA⑥	1	−42.72	C-O⋯H-O	−0.580⋯0.524	0.1873	175.0
SDM-AA⑦	1	−34.64	Pyrimidine para-N⋯H-O	−0.695⋯0.521	0.2012	155.7
SDM-AA⑧	1	−41.59	C-O⋯H-O	−0.587⋯0.518	0.1860	161.4
SDM-EMA①	1	−37.95	N-H⋯O=C	−0.616⋯0.429	0.2009	173.9
SDM-EMA②	1	−38.33	N-H⋯O=C	−0.611⋯0.427	0.2023	173.3
SDM-EMA③	1	−65.29	N-H⋯O=C	−0.638⋯0.460	0.2008	151.6
SDM-EMA④	1	−52.51	N-H⋯O-C	−0.608⋯0.460	0.2015	162.4

* Only strong hydrogen bonds formed by N and O atoms are counted.

**Table 2:** The EBN and HBN_Max_ between several monomers and SDM.

	Functional Monomers						
	AA	MAA	TFMAA	VBA	EHMA	EMA	MMA
**EBN**	2	2	2	2	2	1	2
**HBN_Max_**	3	3	3	3	2	1	2

## Data Availability

Data will be made available on request.

## Supplementary Materials

The following supporting information can be downloaded at https://www.mdpi.com/article/10.3390/molecules29174236/s1, Table S1: Electron donor/acceptor atoms and optimized conformations of the template and functional monomers. Table S2: The chemical structure of sulfonamides; Figure S1: Possible complex formed between SDM and AA at the mole ratio of 1:1. (The dotted red lines are hydrogen bonds); Figure S2: Structures of SDM-TFMAA⑤, SDM-4-VBA⑤and SDM-MAA⑤. (The dotted red lines are hydrogen bonds); Figure S3: Possible complex formed between SDM and EMA at the mole ratio of 1:1. (The dotted red lines are hydrogen bonds); Figure S4: Thermogravimetric curves of SDM and products of each stage; Figure S5: The complexes formed between SDM and AA and TFMAA at the mole ratio of 1:2; Figure S6: The complexes formed between SPA and “holes” of SDM-AA and SDM-TFMAA SMIPs.
