# Supplementary material for: Computational and Experimental Comparison of Molecularly Imprinted Polymers Prepared by Different Functional Monomers—Quantitative Parameters Defined Based on Molecular Dynamics Simulation
Source: Molecules. 2024 Sep 6;29(17):4236. doi: 10.3390/molecules29174236 (PMC11397232; doi:10.3390/molecules29174236)
Supplement: Supplementary file 1 [file molecules-29-04236-s001.zip › molecules-3167831-supplementary.pdf]

Table S1 Electron donor/acceptor atoms and optimized conformations of the template and functional monomers

Abbreviation of chemical name	Chemical structure and hydrogen bond donor (○) or acceptor(○)	The NBO charge of hydrogen bond donor		The NBO charge of hydrogen bond acceptor		Optimized conformation
SDM	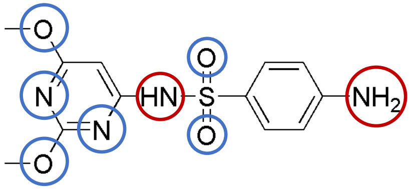	17N-18H	-0.864	15O	-0.918	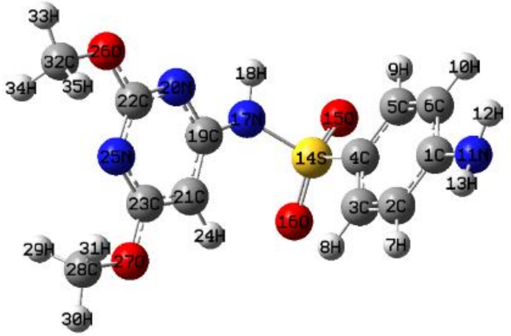
			+0.432	16O	-0.931	
		11N-12H	-0.798	27O	-0.534	
			+0.387	25N	-0.588	
		11N-13H	-0.798	26O	-0.536	
			+0.386	20N	-0.564	
AA	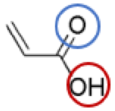	3O-4H	-0.714	2O	-0.598	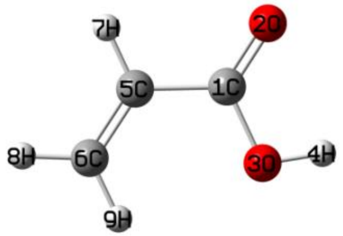
			+0.495			

Abbreviation of chemical name	Chemical structure and hydrogen bond donor (○) or acceptor(○)	The NBO charge of hydrogen bond donor		The NBO charge of hydrogen bond acceptor		Optimized conformation
TFMAA	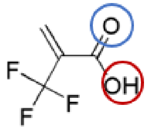	3O-4H	-0.708<br>+0.501	5O	-0.572	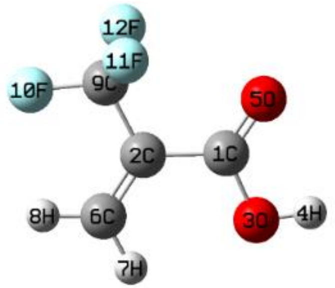
4-VBA	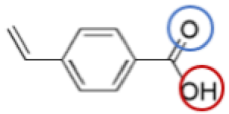	18O-19H	-0.710<br>+0.495	17O	-0.611	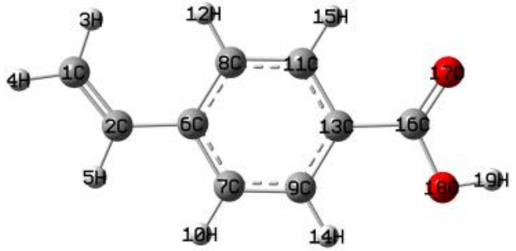
MAA	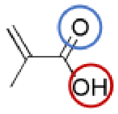	11O-12H	-0.710<br>+0.493	10O	-0.607	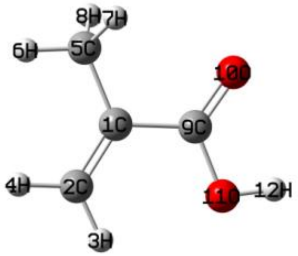

<table>
<tr><th>Abbreviation of chemical name</th><th>Chemical structure and hydrogen bond donor (○) or acceptor(○)</th><th>The NBO charge of hydrogen bond donor</th><th colspan="2">The NBO charge of hydrogen bond acceptor</th><th>Optimized conformation</th></tr>
<tr><td rowspan="2">EMA</td><td rowspan="2">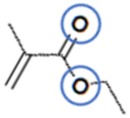</td><td rowspan="2">/</td><td>11O</td><td>-0.570</td><td rowspan="2">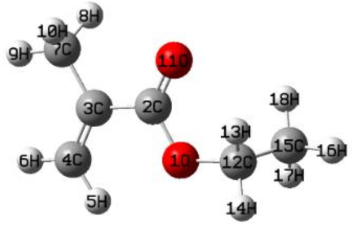
</td></tr>
<tr><td>1O</td><td>-0.587</td></tr>
<tr><td rowspan="2">EHMA</td><td rowspan="2">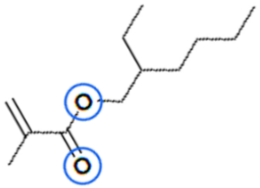</td><td rowspan="2">/</td><td>3O</td><td>-0.590</td><td rowspan="2">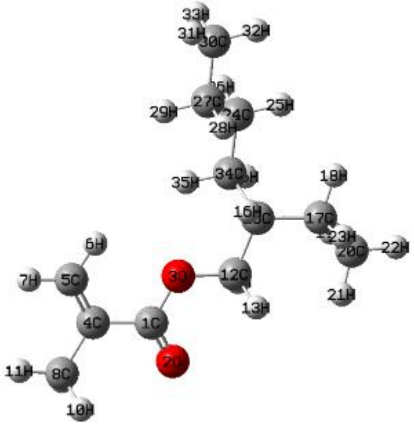</td></tr>
<tr><td>2O</td><td>-0.572</td></tr>
</table>

Abbreviation of chemical name	Chemical structure and hydrogen bond donor (○) or acceptor(○)	The NBO charge of hydrogen bond donor	The NBO charge of hydrogen bond acceptor		Optimized conformation
MMA	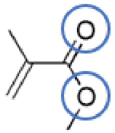	/	11O<br>5O	-0.606<br>-0.575	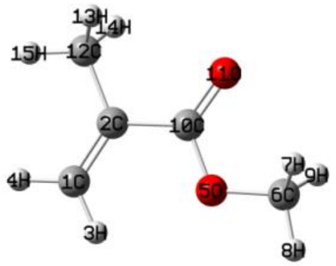

1） SDM-AA①

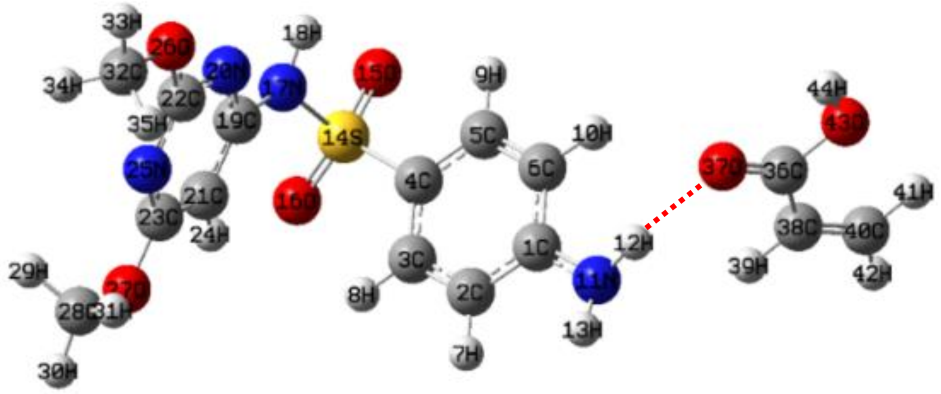

2） SDM-AA②

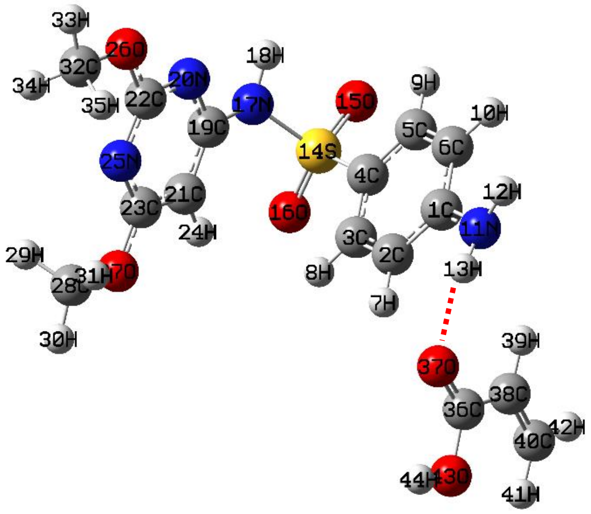

3） SDM-AA③

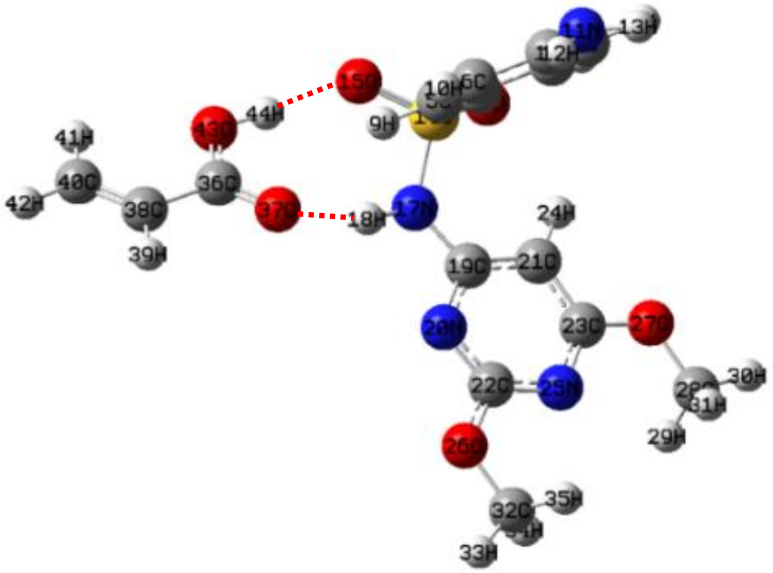

4）SDM-AA④

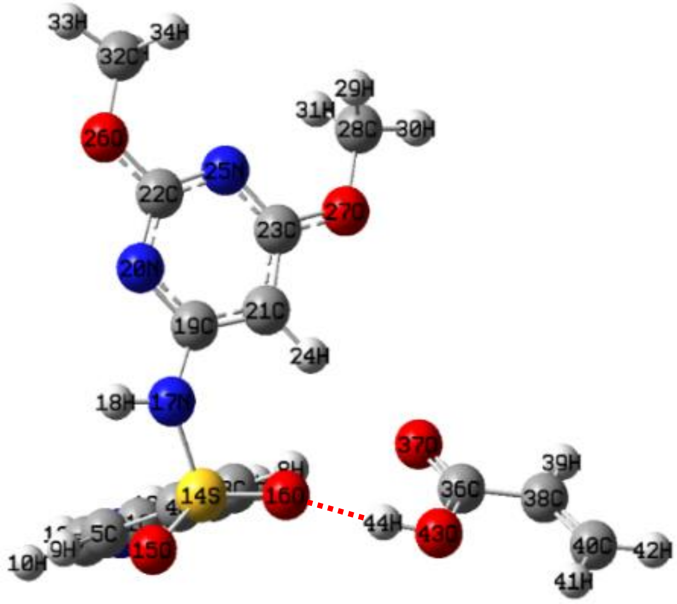

5）SDM-AA⑤

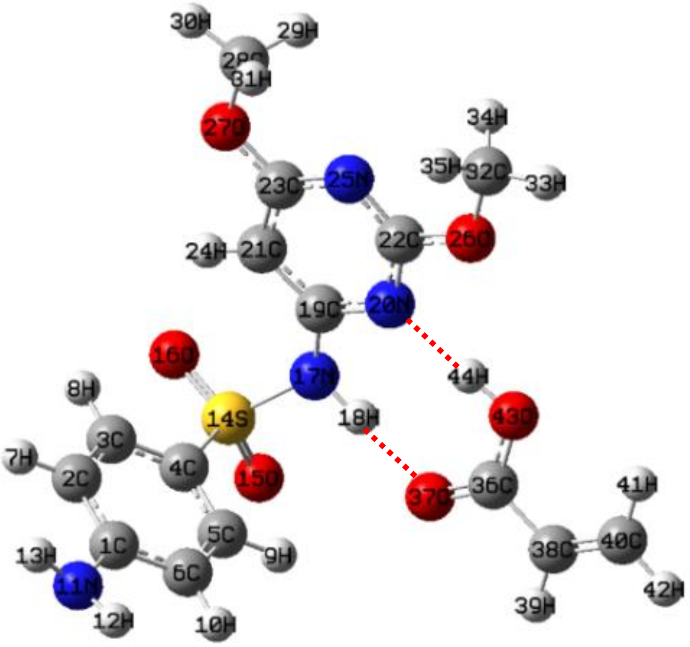

6）SDM-AA⑥

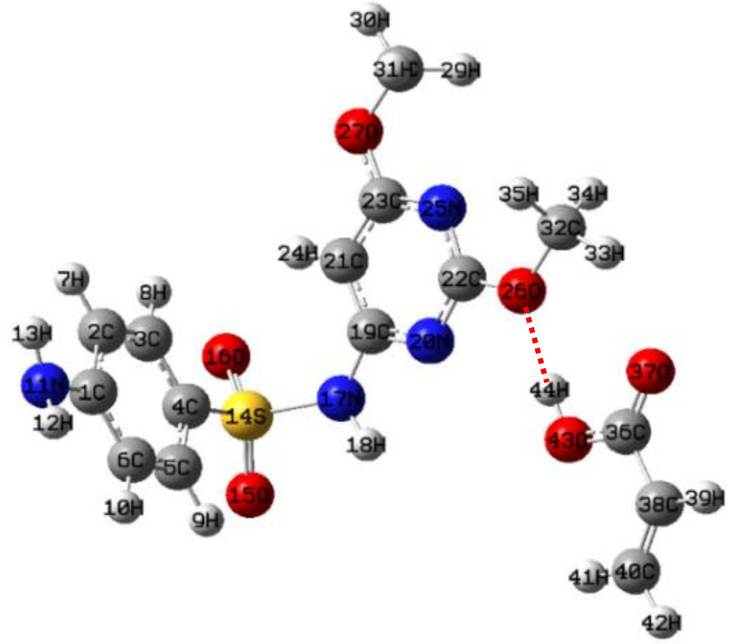

7）SDM-AA⑦

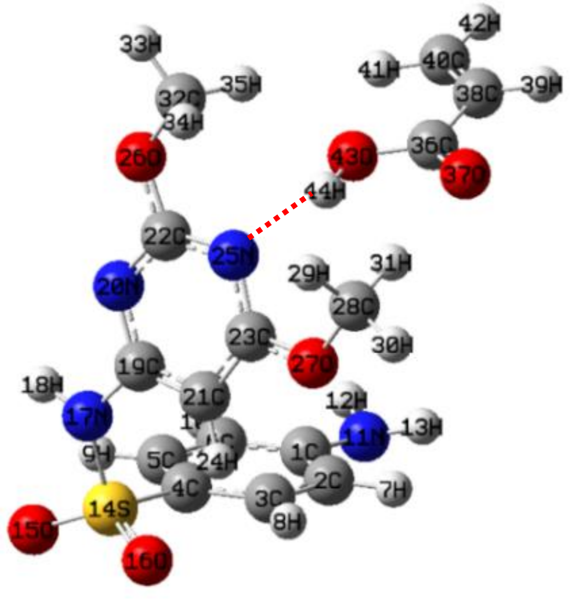

8）SDM-AA⑧

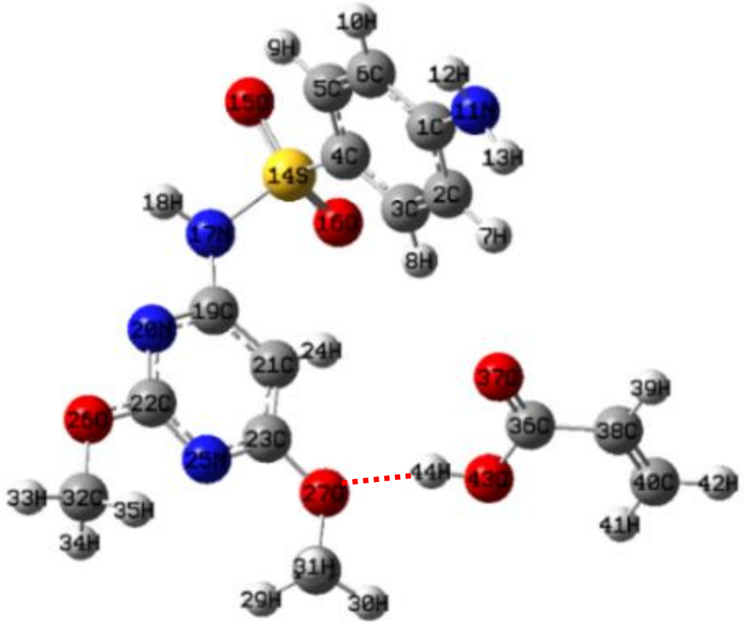

Figure S1. Possible complex formed between SDM and AA at the mole ratio of 1:1.
(The dotted red lines are hydrogen bonds)

1）SDM-TFMAA⑤

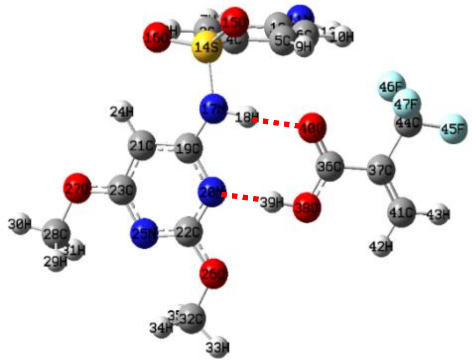

2）SDM-4-VBA⑤

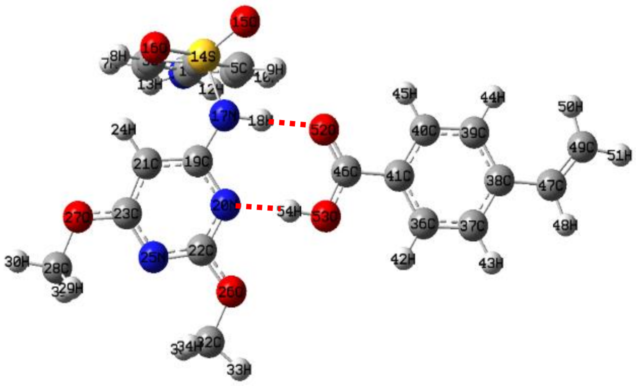

3）SDM-MAA⑤

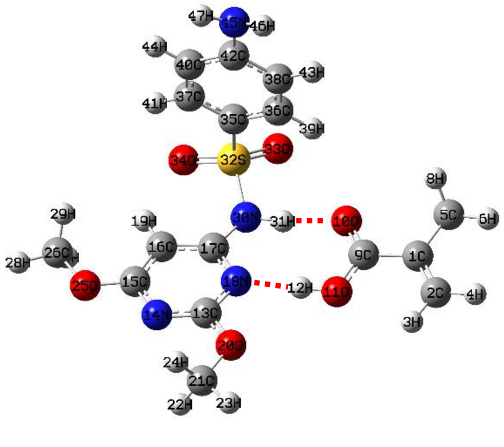

Figure S2. Structures of SDM-TFMAA⑤, SDM-4-VBA⑤and SDM-MAA⑤.
(The dotted red lines are hydrogen bonds)

1)SDM-EMA①

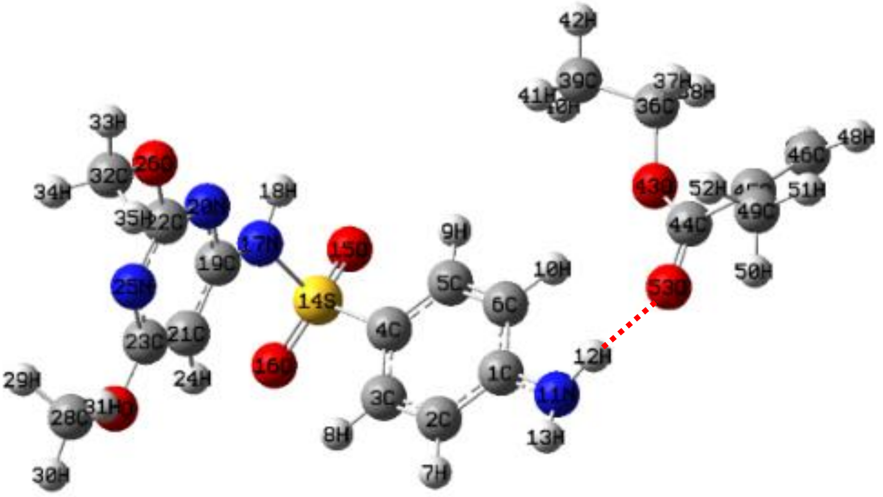

2)SDM-EMA②

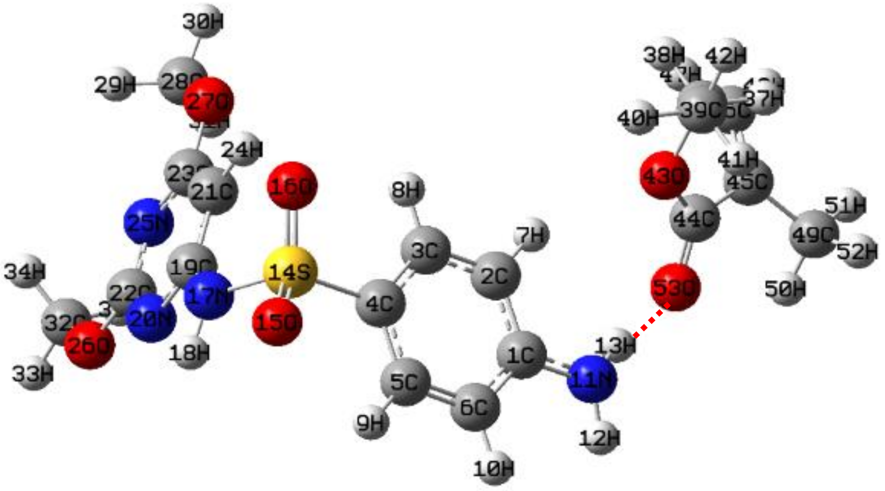

3)SDM-EMA③

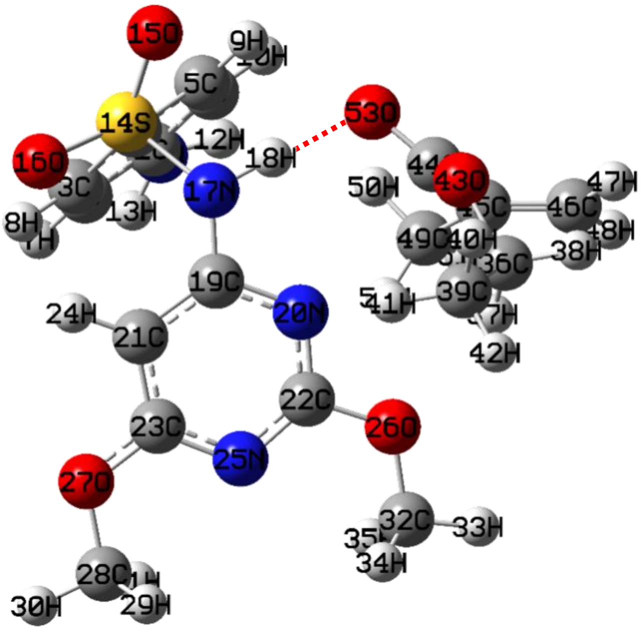

4)SDM-EMA④

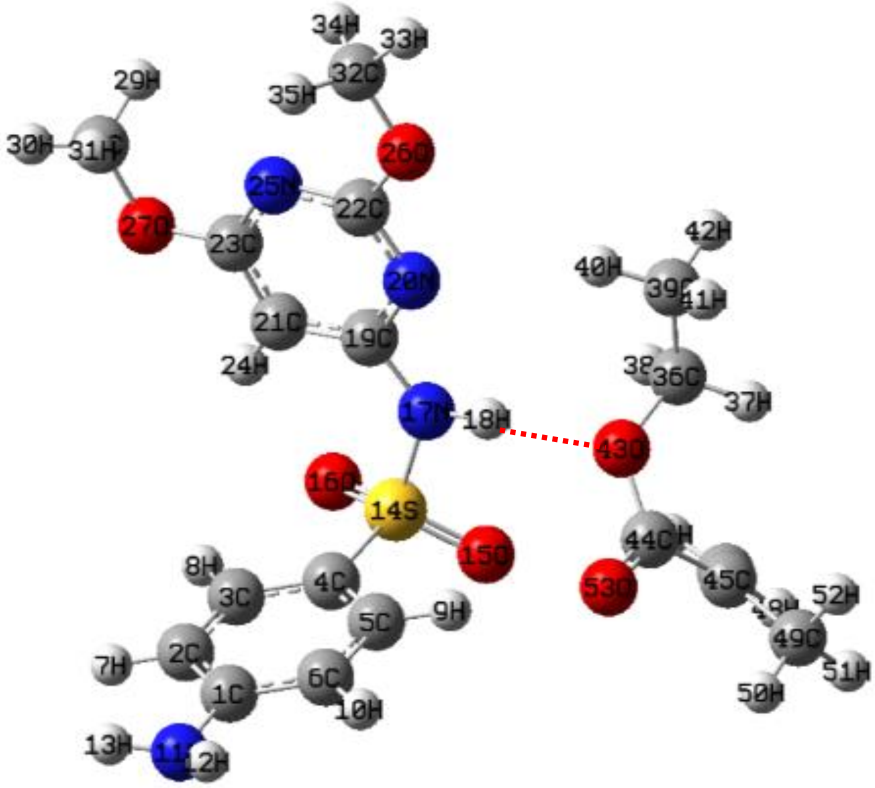

Figure S3. Possible complex formed between SDM and EMA at the mole ratio of 1:1.
(The dotted red lines are hydrogen bonds)

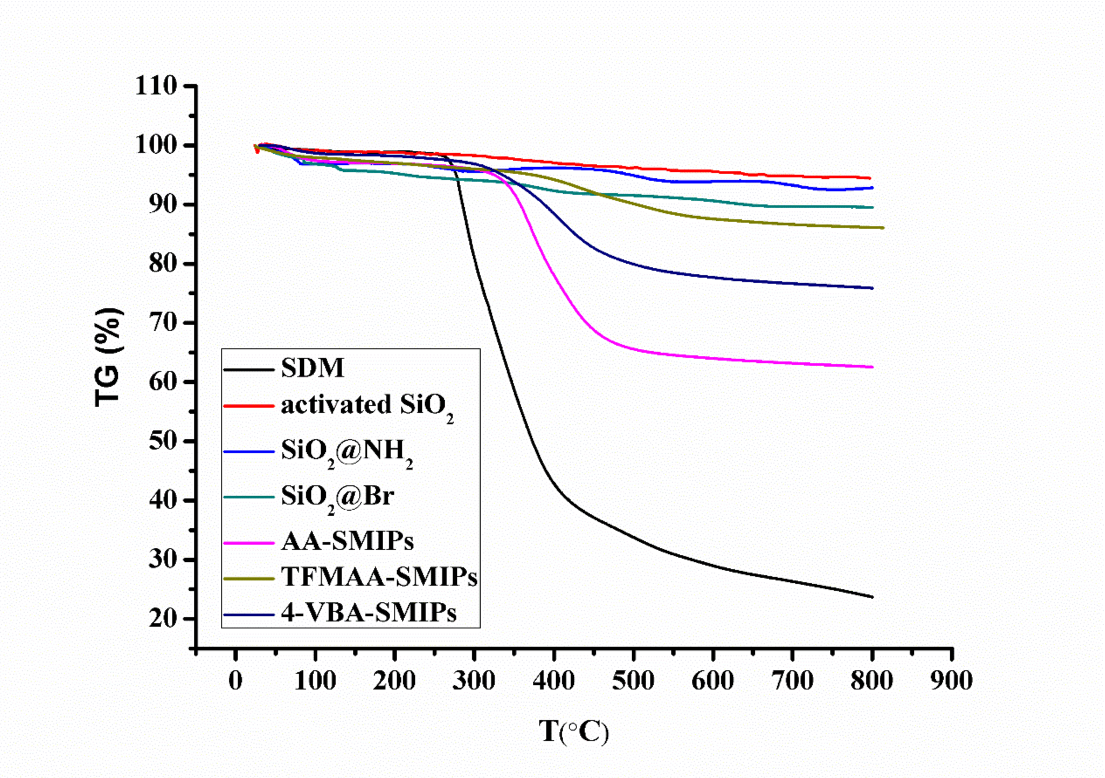

Figure S4 Thermogravimetric curves of SDM and products of each stage.

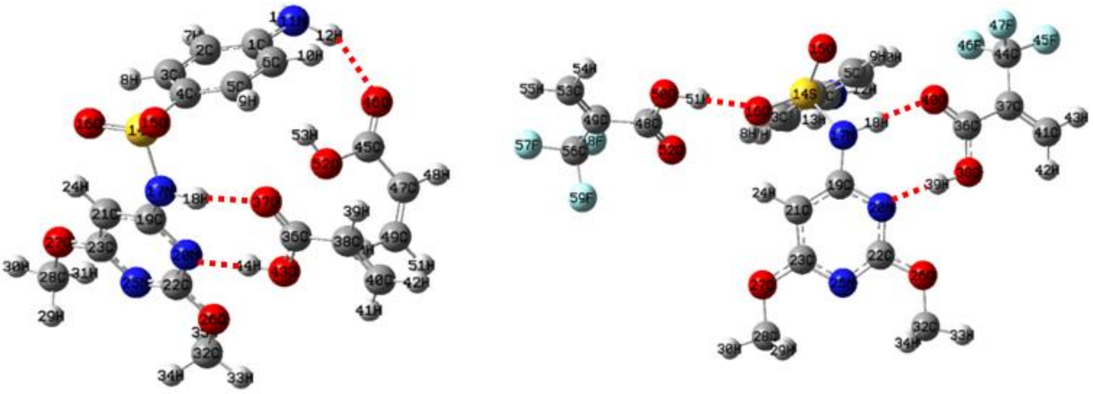

SDM-AA

SDM-TFMAA

Figure S5 The complexes formed between SDM and AA and TFMAA at the mole ratio of 1:2.

Table S2 The chemical structure of sulfonamides.

Drug	Chemical structure	Drug	Chemical structure
SD	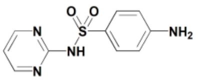	SMP	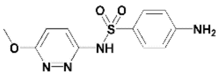
SM2	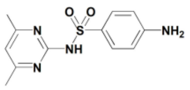	SPA	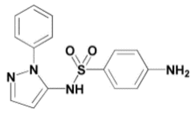
SMM	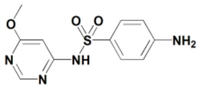	SPD	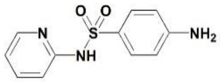

AA

TFMAA

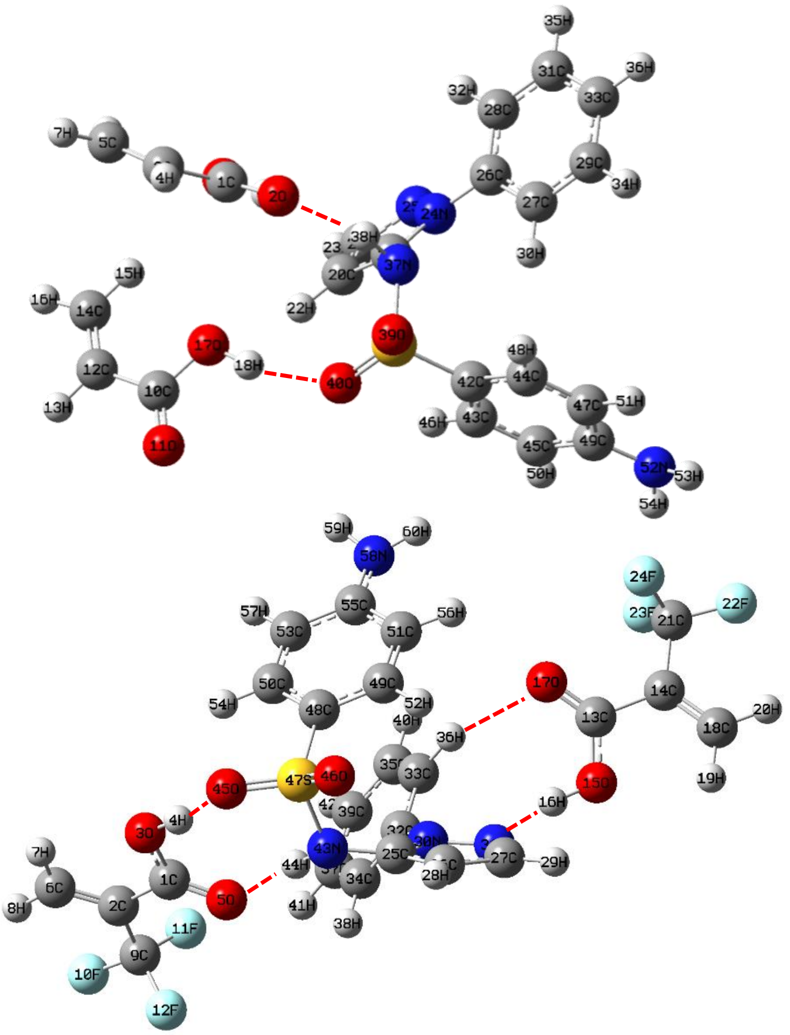

Figure S6 The complexes formed between SPA and “holes” of SDM-AA and SDM-TFMAA SMIPs.
